# Advances in resuscitation and deresuscitation

**DOI:** 10.1097/MCC.0000000000001267

**Published:** 2025-04-21

**Authors:** Olivier Pantet, François-Xavier Ageron, Tobias Zingg

**Affiliations:** a Department of Intensive Care Medicine; b Department of Emergency Medicine; c Department of Visceral Surgery, Lausanne University Hospital – CHUV and University of Lausanne, Lausanne, Switzerland

**Keywords:** balanced crystalloids, deresuscitation techniques, goal-directed fluid therapy, hemodynamic monitoring, resuscitation strategies

## Abstract

**Purpose of review:**

This review aims to provide a perspective on fluid resuscitation strategies and emerging trends in deresuscitation, with a particular emphasis on fluid stewardship, monitoring, and personalized fluid management.

**Recent findings:**

Recent studies underscore a paradigm shift in resuscitation strategies. Notably, aggressive plasma volume expansion has been linked to higher morbidity and mortality, favoring conservative fluid resuscitation. Dynamic parameters, such as pulse pressure variation (PPV) and stroke volume variation (SVV) outperform static markers like central venous pressure (CVP) in predicting preload responsiveness. Advances in hemodynamic monitoring and automated closed-loop fluid administration demonstrate efficacy in optimizing resuscitation. Fluid stewardship, supported by machine learning, is reshaping deresuscitation practices, and promoting negative fluid balance to reduce complications. Moreover, next-generation closed-loop systems and fluid management personalization as part of precision medicine are emerging as future directions.

**Summary:**

Advances in fluid resuscitation challenge traditional practices, with evidence favoring personalized and goal-directed strategies. Technological innovations in hemodynamic monitoring, automated fluid control, and machine learning are driving precision fluid management. Fluid stewardship and deresuscitation aim to mitigate fluid accumulation syndrome and improve patient outcomes.

## INTRODUCTION

**FIGURE 1:**
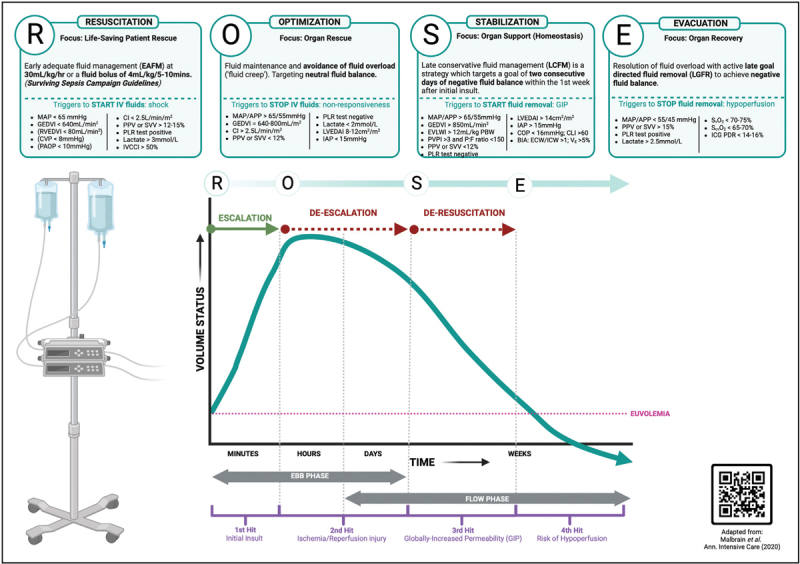
The R(esuscitation) O(ptimization) S(tabilization) E(vacuation) concept.

Resuscitation and deresuscitation are core principles in critical care medicine. Resuscitation focuses on restoring adequate tissue perfusion and oxygenation, while deresuscitation aims to remove excess fluid to prevent complications such as pulmonary edema, abdominal compartment syndrome, and multiorgan dysfunction [1^▪^]. Traditional fluid resuscitation strategies that relied on liberal plasma volume expansion, have evolved into more refined, patient-centered approaches that align fluid administration with preload responsiveness. This shift has been driven by advances in hemodynamic monitoring, the development of personalized fluid titration protocols, and an enhanced understanding of injury and sepsis pathophysiologic responses, that support shift towards precision medicine [2^▪^]. The ROSE concept (Resuscitation, Optimization, Stabilization and Evacuation) clearly depicts the phases of fluid therapy (Fig. 1) for a patient with critical illness or injury [3]. This review summarizes recent fluid management advances that support precise titration of plasma volume expansion to avoid excess total body salt and water, minimize organ injury, and enable more rapid and effective deresuscitation by the intensive care specialist. 

**Box 1:**
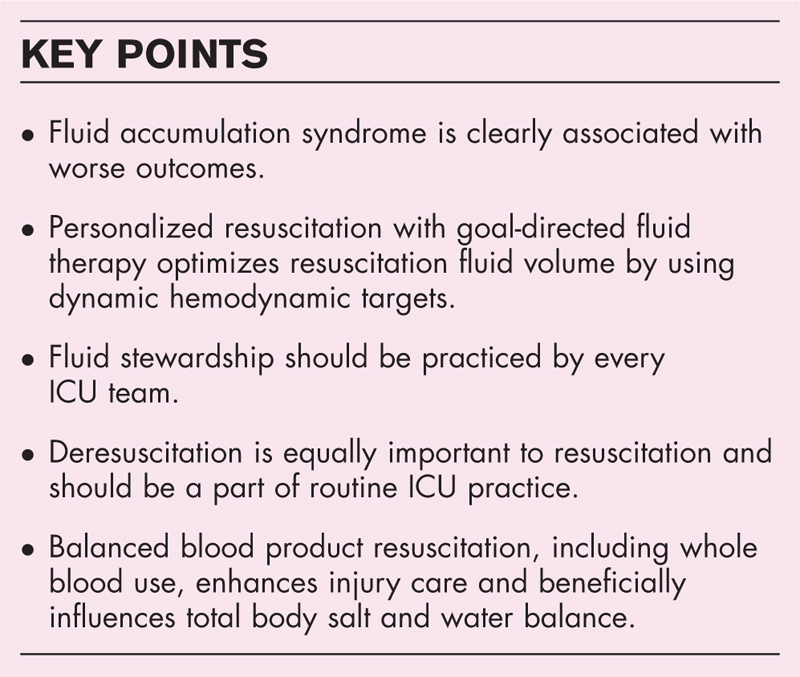
no caption available

## GOAL-DIRECTED FLUID THERAPY

Goal-directed fluid therapy (GDFT) is a cornerstone of modern resuscitation, optimizing cardiac output and oxygen delivery using real-time hemodynamic targets including stroke volume, cardiac output, pulse pressure variation (PPV), and stroke volume variation (SVV). Regardless of the monitoring device, the foundational aspect is that patients should only undergo plasma volume expansion when they demonstrate the potential for preload responsive cardiac performance. This tenet directs clinicians away from treating every episode of hypotension, narrowed pulse pressure, or oliguria as a clear indicator for additional crystalloid infusion [4]. While those with hemorrhagic shock clearly benefit from hemostatic resuscitation ahead of hemorrhage control, those without active hemorrhage benefit from specific evaluation to determine whether plasma volume expansion or a vasoactive agent – of both – is most appropriate [5^▪^,^6^^▪▪^].

The RELIEF and FENICE studies highlighted the benefits of individualized fluid management as well as the wide variability in fluid challenge practices – often without measures of fluid responsivity – particularly in major abdominal surgery and septic shock patients [7,8]. A randomized clinical trial addressing acute pancreatitis demonstrated that unbridled resuscitation resulted in a greater incidence of fluid accumulation without clinical outcome benefit [9]. Soon thereafter, three meta-analyses of pancreatitis care confirmed the accelerated morbidity of aggressive fluid administration [10^▪^,^11^,12^▪^].

Current Surviving Sepsis Campaign (SSC) guidelines recommend dynamic over static parameters to guide fluid administration [13]. A meta-analysis of 69 prospective and interventional studies evaluated five fluid responsiveness assessment maneuvers in invasively ventilated patients. It found PPV, SVV, and plethysmographic variability index (PVI) to be more predictive than CVP or inferior vena cava variation [9]. Additionally, a passive leg raise (PLR), end-expiratory occlusion, or tidal volume challenge also demonstrated reliability [14^▪^]. *Posthoc* analysis of 777 CLASSIC trial patients comparing restrictive and standard fluid strategies found no difference in hyperlactatemia resolution time, even after covariate adjustments [15^▪^]. While another meta-analysis on intraoperative goal-directed hemodynamic therapy (GDHT) using stroke volume optimization found no impact on postoperative complications, acute kidney injury, or mortality, GDHT was associated with reduced hospital length-of-stay. Hydroxyethyl starch reduced, while crystalloids increased postoperative complications perhaps reflecting decreased total body salt and water that could impact pulmonary compliance and anastomotic integrity [16].

Noninvasive hemodynamic monitoring advancements enable real-time GDFT. Automated closed-loop fluid administration systems, which adjust infusion rates in response to continuous feedback, show promise in personalizing fluid volume [17,18]. A RCT of 160 patients compared GDFT with balanced crystalloids versus colloids guided by stroke volume and SVV in conjunction with maintenance crystalloid infusion. Colloid resuscitation led to a lower Post-Operative Morbidity Survey score and fewer complications, likely due to reduced intraoperative fluid volume [19]. An animal model explored the efficacy of computer-controlled algorithmic fluid, vasopressor, and inotrope administration based on dynamic hemodynamic parameters. The system effectively stabilized hemorrhagic shock and treated acute air embolism-induced right heart failure; algorithm performance matched that of expert-guided resuscitation [20^▪^].

One must recognize that bolus fluid for plasma volume expansion is only a small part of the fluid bioburden for the critically ill. Indeed, most fluids are given for nutrition (33%), drug administration (33%) and maintenance (25%) instead of resuscitation (6%) [21]. Accordingly, a pilot study demonstrated that fluid volume can be substantially decreased by protocolized minimization of nonresuscitation fluids [22^▪^].

## RESUSCITATION SOLUTIONS

The landmark SMART and SALT-ED trials established the benefit of balanced crystalloids over 0.9% NaCl in reducing acute kidney injury (AKI), urinary AKI biomarker (N-galactose amine, N-GAL) and composite outcome of mortality, kidney replacement therapy or persistent kidney injury [23,24]. Balanced solutions (e.g. Lactated Ringer's, Normosol-R, and Plasma-Lyte) better match plasma composition and reduce hyperchloremic metabolic acidosis (HCMA) risk. A systematic review and meta-analysis of six studies (34 685 ICU patients) compared balanced crystalloids to 0.9% NaCl suggesting that balanced solutions reduce in-hospital mortality except in those with traumatic brain injury (TBI) [25^▪▪^]. A recent RCT found no differences in mortality, AKI, or six-month neurological outcomes between patients receiving 0.9% NaCl versus a balanced solution; the study was, however, underpowered to establish safety [26]. In hemorrhagic shock, a meta-analysis noted reduced complications (e.g. ARDS, MSOF, AKI) with bicarbonate ringer solution resuscitation [27]. With sepsis, 0.9% NaCl versus Ringer's acetate did not affect major adverse kidney events but appeared to prolong invasive mechanical ventilation duration, perhaps tied to HCMA [28]. Therefore, recent guidelines recommend buffered resuscitation solutions, except in patients with TBI [13,29^▪▪^].

Colloids have been leveraged for their fluid-sparing effect. A meta-analysis of RCTs comparing albumin (4%, 5%, 20%) to crystalloid in sepsis and septic shock noted reduced 90-day mortality with 20% albumin [30]. The SSC guideline suggests using albumin following large volume resuscitation to reduce total salt and water bioburden [13]. Ongoing UK efforts to conduct a randomized controlled trial comparing balanced crystalloids to 5% human albumin in septic patients has demonstrated feasibility but not benefit [31^▪^]. After cardiac surgery, albumin reduced positive fluid balance but without increasing vasopressor infusion duration [32^▪^]. Therefore, albumin seems ideally suited as an adjunct to reduce total body salt and water burden after resuscitation, or for certain patients with hepatic cirrhosis [29^▪▪^,^33^^▪▪^]. Hydroxyethyl starch (HES) use has functionally vanished after the 2014 6S trial noted AKI induction in those with sepsis [34]. Nonetheless, two HES RCTs remain outstanding: TETHYS in trauma [35], and PHOENICS [36] in elective abdominal surgery.

## BLOOD PRODUCT RESUSCITATION

RCTs on prehospital administration of blood products (RBCs and plasma) have yielded mixed results, with unclear survival benefits compared to 0.9% NaCl [37]. This trial, however, did not include platelets in the component therapy arm, and did not occur in a system with short transport time, thereby confounding the impact of component therapy on outcome and time to hemorrhage control. The reintroduction of low-titer O+ whole blood (LTOWB) in civilian trauma care has substantially increased in prehospital settings and provides hemostatic resuscitation that includes platelets [5^▪^]. A single-center RCT comparing whole blood to component therapy in patients requiring massive transfusion found similar overall transfusion volumes. However, excluding severe brain injury cases, whole blood significantly reduced 24-h RBCs, plasma, platelets, and total blood product volumes [38]. LTOWB simplifies logistics – especially for aeromedical rescue – shortens transfusion times and offers survival benefits in severe hemorrhagic shock and during military conflict where hemostatic resuscitation is challenging [39^▪▪^,40^▪^].

Advances in trauma-induced coagulopathy (TIC) knowledge and management support balanced transfusion strategies that prioritize early component therapy transfusion or whole blood augmented by agents such as tranexamic acid (TXA) to support adaptive thrombosis. The PATCH trial demonstrated that early prehospital TXA administration was safe, effective, and reduced mortality but did not improve longer-term functional outcome within an advanced trauma system [41,42^▪^]. Other conditions associated with hemorrhagic shock are also beneficially impacted by TXA bolus and infusion. TXA is recommended for postpartum hemorrhage (PPH) to reduce hemorrhage-associated mortality without maladaptive thrombosis occurrence [43^▪▪^]. TXA reduced life-threatening bleeding and major bleeding at 30-days in noncardiac surgery patients [44]. It is plausible that cryoprecipitate or Prothrombin Complex Concentrate (PCC) may improve outcomes following injury. Unfortunately, CRYOSTAT-2 found no benefit in injured patients [45^▪▪^]. Similarly, the PROCOAG trial failed to find any impact on component therapy requirements following empiric postinjury treatment with PCC [46^▪^].

## RESUSCITATION ADJUNCTS

Resuscitative Endovascular Balloon Occlusion of the Aorta (REBOA) has gained traction over the past decade, but its impact on morbidity and mortality remains uncertain. A pragmatic RCT at 16 UK trauma centers compared REBOA plus standard care (*n* = 46) to standard care (*n* = 44) in 90 patients with exsanguinating hemorrhage. The REBOA group showed higher 90-day mortality [54% versus 42%, odds ratio (OR) 1.58, 95% confidence interval (CI) 0.72–3.52] and an 86.9% probability that REBOA increases 24-h mortality, suggesting harm in this patient population [47^▪▪^].

Point-of-care viscoelastic tests (VET), such as thromboelastography (TEG) and rotational thromboelastometry (ROTEM), facilitate rapid coagulation assessment to guide individualized transfusion and reduce unnecessary component use during resuscitation [48]. While the original ITACTIC trial [49] failed to discern a difference in care guided by VET, a secondary analysis revealed that despite earlier treatment in the VET group, goal-directed interventions were often delayed, leading to incomplete coagulopathy correction and delayed therapy [50^▪^]. Accurate identification of deficits in clotting factors, platelets, calcium, as well as the presence of hypercoagulability or fibrinolysis establish VET as a key advance in injury care [51^▪^].

## PHARMACOLOGIC ADJUNCTS TO RESUSCITATION

The early and judicious use of vasopressors is essential in resuscitating shock. The CLOVERS trial, an unblinded superiority trial at 60 US centers randomized 1563 sepsis-induced hypotension patients to either a restrictive fluid strategy (prioritizing vasopressors) or a liberal fluid strategy (higher IV fluid volumes) for 24 h. The restrictive strategy did not significantly reduce mortality before discharge by day 90 (14.0% versus 14.9%; *P* = 0.61), with comparable serious adverse event rates between groups [52^▪▪^]. A posthoc analysis of the CLOVERS trial including 196 patients with advanced chronic kidney disease demonstrated a lower mortality at 90 days in the early restrictive strategy group [53^▪^].

## DERESUSCITATION

Deresuscitation refers to the active removal of excess fluid following the resuscitation phase to counter the complications of positive fluid balance, which is linked to higher mortality in critically ill patients. The maladaptive excess of fluid volume (and salt) that adversely impacts organ function is termed ‘fluid accumulation syndrome’ [54]. Well studied in sepsis and septic shock, the notion of aggressive fluid administration driven anasarca is linked to injury care, intra-abdominal hypertension, and the abdominal compartment syndrome [55]. Therefore, approaches that limit crystalloid resuscitation, such as hemostatic resuscitation, are coupled with planned fluid removal – instead of awaiting spontaneous mobilization of excess salt and water. Deresuscitation firmly establishes planned reductions in salt and water as the ‘E’ phase of the ROSE approach. Protocolized reduced fluid infusion rates, diuretic therapy, and kidney replacement therapy (KRT) – both intermittent and continuous – may be utilized for deresuscitation. Concerns regarding engaging in deresuscitation include creating recurrent hypovolemia and hypoperfusion as well as new organ injury [56]. To date, none of these concerns demonstrate validity, especially since many trials fail to achieve substantial fluid volume separation between treated and untreated groups. Accordingly, results using different deresuscitation techniques in disparate trials are heterogeneous.

The RADAR-2 trial tested the feasibility of active fluid removal. The intervention significantly reduced 24-h and cumulative fluid balance but was not powered to assess patient-centered outcomes [57]. More recently, the POINTCARE-2 study, a stepped wedge cluster open-label RCT failed to demonstrate reduced 60-day mortality; its methods however, did not ensure that there was exposure to the fluid reduction strategy leaving many questions unanswered [58^▪^]. Similarly, a systematic review found no significant mortality reduction or notable fluid balance or patient-centered outcome impact with active deresuscitation versus usual care [70]. Small sample sizes and methodological issues – including a lack of fluid volume separation between groups – established major limitations [1^▪^]. Another major problem in assessing the impact of deresuscitation is the heterogeneity of criteria that identifies a patient appropriate for planned salt and water loss including an assessment of preload dependence. While there are many assessments that may be deployed, the venous congestion by point-of-care-ultrasound (VExUS) score seems to strongly correlate with invasively measured cardiac filling pressures [59^▪^]. The difference of VExUS scores between day 1 and day 3 is associated a composite major adverse kidney event metric, but not with AKI nor mortality as unique endpoints [60]. The data suggest that an increased VExUS score is a reasonable trigger for deresuscitation.

## FLUID REMOVAL TECHNIQUES

Loop diuretics, (i.e. furosemide) remain the primary agents for deresuscitation either as monotherapy or in combination with spironolactone, thiazides, or acetazolamide. In fluid-overloaded patients requiring KRT, a goal-directed, perfusion-based deresuscitation strategy using continuous KRT (CKRT) achieved a greater cumulative negative fluid balance without inducing hemodynamically compromise versus standard practice [61]. Importantly, this study achieved what many did not – fluid volume separation between cohorts. Currently, the selection of KRT mode remains driven by patient factors rather than by mode characteristics. While randomized trials show no mortality benefit of CKRT over intermittent hemodialysis, CKRT enables dynamic, personalized treatment, and may promote better renal recovery and KRT independence in critically ill patients [62^▪^].

## EMERGING CONCEPTS IN FLUID MANAGEMENT

Achieving appropriate fluid balance is a key goal in critical care. Much like Antibiotic Stewardship Programs help guide appropriate use of antimicrobials [63^▪^], a similar approach is suitable for fluid stewardship [64^▪^]. Fluid stewardship includes evaluating maintenance fluids as well as therapeutic agents as contributors to total balance [65,66]. The PharmD is a key team member in addressing fluid stewardship, and PharmD stewardship interventions seem to correlate with medication regimen complexity [67]. Relatedly, a multicenter (31 ICUs in France and Spain) analysis of fluid volume, type, and intent (i.e. homeostasis, resuscitation, etc.) at 24 h noted that fluids that supported homeostasis were the smallest contributor to fluid balance [68^▪^]. Unsurprisingly, the main determinant of total fluid balance was the center – suggesting that local practice patterns, rather than evidence, guide how nonhomeostasis fluids are administered. The emergence of machine learning/augmented intelligence tools embedded in the electronic health record could provide ‘nudges’ to clinicians to address fluid balance once certain thresholds are breached [69^▪^]. In this way, decision-support tools may aid clinicians in rigorously monitoring cumulative balance, minimizing unnecessary fluid administration, and triggering appropriate deresuscitation [64^▪^]. Such tools may be linked with closed-loop fluid management systems that are integrated with continuous hemodynamic monitoring to autonomously regulate fluid administration that is titrated to a prespecified goal [70]. Current research aims to individualize fluid management based on genetic, metabolic, and hemodynamic profiles. Advances in ’omics’ technologies, including metabolomics and proteomics, may enable personalized resuscitation protocols in the future [2^▪^].

## CONCLUSION

Advancements in fluid management, spanning resuscitation to deresuscitation continue to refine critical care practices. The shift from static to dynamic resuscitation, Goal-Directed Fluid Therapy (GDFT) underscores a continued move towards a precision-driven approach. Balanced crystalloids, selective colloid use, and novel blood product strategies aim to optimize patient outcomes. Increasingly embraced practices such as fluid stewardship and deresuscitation address the deleterious effects of fluid accumulation syndrome to reduce morbidity and mortality. Technological innovations, including ML/AI-driven prediction models are shaping the future of individualized fluid management to ensure an increasingly efficient – and patient-centered – approach to fluid therapy during critical illness.

## Acknowledgements

*The authors thank Dr Gary Bass, MD, MSc, MBA, PhD, FEBS EmSurg for the illustration used inFig. 1*.

### Financial support and sponsorship

*No financial support or sponsorship was received for the purpose of this article.*

### Conflicts of interest

*There are no conflicts of interest.*

## REFERENCES AND RECOMMENDED READING

Papers of particular interest, published within the annual period of review, have been highlighted as:

▪ of special interest
▪▪ of outstanding interest

## References

[1▪] Messmer AS Dill T Müller M. Active fluid de-resuscitation in critically ill patients with septic shock: a systematic review and meta-analysis. Eur J Intern Med 2023; 109:89–96. 36635127 10.1016/j.ejim.2023.01.009

[2▪] Gordon AC Alipanah-Lechner N Bos LD. From ICU syndromes to ICU subphenotypes: consensus report and recommendations for developing precision medicine in the ICU. Am J Respir Crit Care Med 2024; 210:155–166. 38687499 10.1164/rccm.202311-2086SO PMC11273306

[3] Malbrain M Martin G Ostermann M. Everything you need to know about deresuscitation. Intensive Care Med 2022; 48:1781–1786. 35932335 10.1007/s00134-022-06761-7 PMC9362613

[4] Hofmaenner DA Singer M. Challenging management dogma where evidence is nonexistent, weak, or outdated: part II. Intensive Care Med 2024; 50:1804–1813. 39320462 10.1007/s00134-024-07634-x

[5▪] Coulthard SL Kaplan LJ Cannon JW. What's new in whole blood resuscitation? In the trauma bay and beyond. Curr Opin Crit Care 2024; 30:209–216. 38441127 10.1097/MCC.0000000000001140

[6▪▪] Monnet X Malbrain M Pinsky MR. The prediction of fluid responsiveness. Intensive Care Med 2023; 49:83–86. 36323911 10.1007/s00134-022-06900-0

[7] Cecconi M Hofer C Teboul JL. Fluid challenges in intensive care: the FENICE study: a global inception cohort study. Intensive Care Med 2015; 41:1529–1537. 26162676 10.1007/s00134-015-3850-x PMC4550653

[8] Myles PS Bellomo R Corcoran T. Restrictive versus liberal fluid therapy for major abdominal surgery. N Engl J Med 2018; 378:2263–2274. 29742967 10.1056/NEJMoa1801601

[9] de-Madaria E Buxbaum JL Maisonneuve P. Aggressive or moderate fluid resuscitation in acute pancreatitis. N Engl J Med 2022; 387:989–1000. 36103415 10.1056/NEJMoa2202884

[10▪] Li XW Wang CH Dai JW. Comparison of clinical outcomes between aggressive and nonaggressive intravenous hydration for acute pancreatitis: a systematic review and meta-analysis. Crit Care 2023; 27:122. 36949459 10.1186/s13054-023-04401-0 PMC10035244

[11] Dawson A Karunakaran M Sharma ZD. Fluid resuscitation in the early management of acute pancreatitis - evidence from a systematic review and meta-analysis. HPB (Oxford) 2023; 25:1451–1465. 37689561 10.1016/j.hpb.2023.08.013

[12▪] Evans D Hajibandeh S Hajibandeh S. Meta-analysis and trial sequential analysis of randomized controlled trials comparing aggressive versus nonaggressive intravenous fluid therapy in acute pancreatitis: an insight into the existence of type 2 error. J Gastroenterol Hepatol 2024; 39:2018–2030. 38872377 10.1111/jgh.16648

[13] Evans L Rhodes A Alhazzani W. Surviving sepsis campaign: international guidelines for management of sepsis and septic shock 2021. Intensive Care Med 2021; 47:1181–1247. 34599691 10.1007/s00134-021-06506-y PMC8486643

[14▪] Chaves RCF Barbas CSV Queiroz VNF. Assessment of fluid responsiveness using pulse pressure variation, stroke volume variation, plethysmographic variability index, central venous pressure, and inferior vena cava variation in patients undergoing mechanical ventilation: a systematic review and meta-analysis. Crit Care 2024; 28:289. 39217370 10.1186/s13054-024-05078-9 PMC11366151

[15▪] Ahlstedt C Sivapalan P Kriz M. Effects of restrictive fluid therapy on the time to resolution of hyperlactatemia in ICU patients with septic shock. A secondary post hoc analysis of the CLASSIC randomized trial. Intensive Care Med 2024; 50:678–686. 38598125 10.1007/s00134-024-07385-9 PMC11078841

[16] Ripollés-Melchor J Espinosa ÁV Fernández-Valdes-Bango P. Intraoperative goal-directed hemodynamic therapy through fluid administration to optimize the stroke volume: a meta-analysis of randomized controlled trials. Rev Esp Anestesiol Reanim (Engl Ed) 2024; 71:719–731. 39243815 10.1016/j.redare.2024.09.004

[17] Alexander B Rinehart J Cannesson M. Closed-loop hemodynamic management. Best Pract Res Clin Anaesthesiol 2019; 33:199–209. 31582099 10.1016/j.bpa.2019.04.003

[18] Avital G Snider EJ Berard D. Closed-loop controlled fluid administration systems: a comprehensive scoping Review. J Pers Med 2022; 12:1168. 35887665 10.3390/jpm12071168 PMC9315597

[19] Joosten A Delaporte A Ickx B. Crystalloid versus colloid for intraoperative goal-directed fluid therapy using a closed-loop system: a randomized, double-blinded, controlled trial in major abdominal surgery. Anesthesiology 2018; 128:55–66. 29068831 10.1097/ALN.0000000000001936

[20▪] Pinsky MR Gomez H Wertz A. Evaluation of a physiologic-driven closed-loop resuscitation algorithm in an animal model of hemorrhagic shock. Crit Care Med 2024; 52:1947–1957. 39436216 10.1097/CCM.0000000000006297

[21] Van Regenmortel N Verbrugghe W Roelant E. Maintenance fluid therapy and fluid creep impose more significant fluid, sodium, and chloride burdens than resuscitation fluids in critically ill patients: a retrospective study in a tertiary mixed ICU population. Intensive Care Med 2018; 44:409–417. 29589054 10.1007/s00134-018-5147-3 PMC5924672

[22▪] Linden A Spangfors M Olsen MH. Protocolized reduction of nonresuscitation fluids versus usual care in septic shock patients (REDUSE): a randomized multicentre feasibility trial. Crit Care 2024; 28:166. 38760833 10.1186/s13054-024-04952-w PMC11100208

[23] Semler MW Self WH Wanderer JP. Balanced crystalloids versus saline in critically ill adults. N Engl J Med 2018; 378:829–839. 29485925 10.1056/NEJMoa1711584 PMC5846085

[24] Self WH Semler MW Wanderer JP. Balanced crystalloids versus saline in noncritically ill adults. N Engl J Med 2018; 378:819–828. 29485926 10.1056/NEJMoa1711586 PMC5846618

[25▪▪] Zampieri FG Cavalcanti AB Di Tanna GL. Balanced crystalloids versus saline for critically ill patients (BEST-Living): a systematic review and individual patient data meta-analysis. Lancet Respir Med 2024; 12:237–246. 38043564 10.1016/S2213-2600(23)00417-4

[26] Poh K Bustam A Hasan MS. Isotonic balanced fluid versus 0.9% saline in patients with moderate to severe traumatic brain injury: A double-blinded randomised controlled trial. Am J Emerg Med 2024; 77:106–114. 38118385 10.1016/j.ajem.2023.11.064

[27] Shafique MA Shaikh NA Haseeb A. Sodium bicarbonate Ringer's solution for hemorrhagic shock: a meta-analysis comparing crystalloid solutions. Am J Emerg Med 2024; 76:41–47. 37988980 10.1016/j.ajem.2023.11.003

[28] Zhang J Liu F Wu Z. Acetate ringer's solution versus normal saline solution in sepsis: a randomized, controlled trial. Shock 2024; 61:520–526. 38369528 10.1097/SHK.0000000000002324

[29▪▪] Arabi YM Belley-Cote E Carsetti A. European Society of Intensive Care Medicine clinical practice guideline on fluid therapy in adult critically ill patients. Part 1: the choice of resuscitation fluids. Intensive Care Med 2024; 50:813–831. 38771364 10.1007/s00134-024-07369-9

[30] Geng L Tian X Gao Z. Different concentrations of albumin versus crystalloid in patients with sepsis and septic shock: a meta-analysis of randomized clinical trials. J Intensive Care Med 2023; 38:679–689. 37078161 10.1177/08850666231170778

[31▪] Gray AJ Oatey K Grahamslaw J. Albumin versus balanced crystalloid for the early resuscitation of sepsis: an open parallel-group randomized feasibility trial- the ABC-Sepsis Trial. Crit Care Med 2024; 52:1520–1532. 38912884 10.1097/CCM.0000000000006348

[32▪] Wigmore GJ Deane AM Presneill JJ. Twenty percentage human albumin solution fluid bolus administration therapy in patients after cardiac surgery. II: a multicentre randomised controlled trial. Intensive Care Med 2024; 50:1075–1085. 38953926 10.1007/s00134-024-07488-3 PMC11245445

[33▪▪] Callum J Skubas NJ Bathla A. Use of intravenous albumin: a guideline from the international collaboration for transfusion medicine guidelines. Chest 2024; 166:321–338. 38447639 10.1016/j.chest.2024.02.049 PMC11317816

[34] Myburgh JA Finfer S Bellomo R. Hydroxyethyl starch or saline for fluid resuscitation in intensive care. N Engl J Med 2012; 367:1901–1911. 23075127 10.1056/NEJMoa1209759

[35] Palma CD Mamba M Geldenhuys J. PragmaTic, prospEctive, randomized, controlled, double-blind, mulTicentre, multinational study on the safety and efficacy of a 6% HydroxYethyl Starch (HES) solution versus an electrolyte solution in trauma patients: study protocol for the TETHYS study. Trials 2022; 23:456. 35655234 10.1186/s13063-022-06390-x PMC9164328

[36] Buhre W de Korte-de Boer D de Abreu MG. Prospective, randomized, controlled, double-blind, multicenter, multinational study on the safety and efficacy of 6% Hydroxyethyl starch (HES) sOlution versus an Electrolyte solutioN In patients undergoing eleCtive abdominal Surgery: study protocol for the PHOENICS study. Trials 2022; 23:168. 35193648 10.1186/s13063-022-06058-6 PMC8862305

[37] Crombie N Doughty HA Bishop JRB. Resuscitation with blood products in patients with trauma-related haemorrhagic shock receiving prehospital care (RePHILL): a multicentre, open-label, randomised, controlled, phase 3 trial. Lancet Haematol 2022; 9:e250–e261. 35271808 10.1016/S2352-3026(22)00040-0 PMC8960285

[38] Cotton BA Podbielski J Camp E. A randomized controlled pilot trial of modified whole blood versus component therapy in severely injured patients requiring large volume transfusions. Ann Surg 2013; 258:527–532. discussion 32-33. 23979267 10.1097/SLA.0b013e3182a4ffa0

[39▪▪] Morgan KM Abou Khalil E Feeney EV. The efficacy of low-titer group O whole blood compared with component therapy in civilian trauma patients: a meta-analysis. Crit Care Med 2024; 52:e390–e404. 38483205 10.1097/CCM.0000000000006244

[40▪] Meizoso JP Cotton BA Lawless RA. Whole blood resuscitation for injured patients requiring transfusion: a systematic review, meta-analysis, and practice management guideline from the Eastern Association for the Surgery of Trauma. J Trauma Acute Care Surg 2024; 97:460–470. 38531812 10.1097/TA.0000000000004327

[41] Guyette FX Brown JB Zenati MS. Tranexamic acid during prehospital transport in patients at risk for hemorrhage after injury: a double-blind, placebo-controlled, randomized clinical trial. JAMA Surg 2020; 156:11–20. 33016996 10.1001/jamasurg.2020.4350 PMC7536625

[42▪] Investigators PA-T the ACTG Gruen RL. Prehospital tranexamic acid for severe trauma. N Engl J Med 2023; 389:127–136. 37314244 10.1056/NEJMoa2215457

[43▪▪] Ker K Sentilhes L Shakur-Still H. Tranexamic acid for postpartum bleeding: a systematic review and individual patient data meta-analysis of randomised controlled trials. Lancet 2024; 404:1657–1667. 39461793 10.1016/S0140-6736(24)02102-0 PMC12197804

[44] Devereaux PJ Marcucci M Painter TW. Tranexamic acid in patients undergoing noncardiac surgery. N Engl J Med 2022; 386:1986–1997. 35363452 10.1056/NEJMoa2201171

[45▪▪] Davenport R Curry N Fox EE. Early and empirical high-dose cryoprecipitate for hemorrhage after traumatic injury: the CRYOSTAT-2 randomized clinical trial. JAMA 2023; 330:1882–1891. 37824155 10.1001/jama.2023.21019 PMC10570921

[46▪] Bouzat P Charbit J Abback PS. Efficacy and safety of early administration of 4-factor prothrombin complex concentrate in patients with trauma at risk of massive transfusion: the PROCOAG randomized clinical trial. JAMA 2023; 329:1367–1375. 36942533 10.1001/jama.2023.4080 PMC10031505

[47▪▪] Jansen JO Hudson J Cochran C. Emergency department resuscitative endovascular balloon occlusion of the aorta in trauma patients with exsanguinating hemorrhage: the UK-REBOA randomized clinical trial. JAMA 2023; 330:1862–1871. 37824132 10.1001/jama.2023.20850 PMC10570916

[48] Zipperle J Schmitt FCF Schochl H. Point-of-care, goal-directed management of bleeding in trauma patients. Curr Opin Crit Care 2023; 29:702–712. 37861185 10.1097/MCC.0000000000001107

[49] Baksaas-Aasen K Gall LS Stensballe J. Viscoelastic haemostatic assay augmented protocols for major trauma haemorrhage (ITACTIC): a randomized, controlled trial. Intensive Care Med 2021; 47:49–59. 33048195 10.1007/s00134-020-06266-1 PMC7550843

[50▪] Lindsay C Davenport R Baksaas-Aasen K. Correction of trauma-induced coagulopathy by goal-directed therapy: a secondary analysis of the ITACTIC trial. Anesthesiology 2024; 141:904–912. 39115454 10.1097/ALN.0000000000005183 PMC11462890

[51▪] Juffermans NP Bouzat P. Visco-elastic testing in traumatic bleeding. Intensive Care Med 2024; 50:1152–1153. 38695918 10.1007/s00134-024-07437-0

[52▪▪] Shapiro NI Douglas IS Brower RG. Early restrictive or liberal fluid management for sepsis-induced hypotension. N Engl J Med 2023; 388:499–510. 36688507 10.1056/NEJMoa2212663 PMC10685906

[53▪] Jorda A Douglas IS Staudinger T. Fluid management for sepsis-induced hypotension in patients with advanced chronic kidney disease: a secondary analysis of the CLOVERS trial. Critical care 2024; 28:231. 38992663 10.1186/s13054-024-05019-6 PMC11238412

[54] Pfortmueller CA Dabrowski W Wise R. Fluid accumulation syndrome in sepsis and septic shock: pathophysiology, relevance and treatment-a comprehensive review. Ann Intensive Care 2024; 14:115. 39033219 10.1186/s13613-024-01336-9 PMC11264678

[55] Smit M van Meurs M Zijlstra JG. Intra-abdominal hypertension and abdominal compartment syndrome in critically ill patients: a narrative review of past, present, and future steps. Scand J Surg 2022; 111:14574969211030128. 34605332 10.1177/14574969211030128

[56] Chacko J Pawar S Seppelt I Brar G. Controversies in critical care. Singapore: Springer Nature; 2023.

[57] Silversides JA McMullan R Emerson LM. Feasibility of conservative fluid administration and deresuscitation compared with usual care in critical illness: the Role of Active Deresuscitation After Resuscitation-2 (RADAR-2) randomised clinical trial. Intensive Care Med 2022; 48:190–200. 34913089 10.1007/s00134-021-06596-8

[58▪] Bollaert PE Monnier A Schneider F. Fluid balance control in critically ill patients: results from POINCARE-2 stepped wedge cluster-randomized trial. Crit Care 2023; 27:66. 36810101 10.1186/s13054-023-04357-1 PMC9945675

[59▪] Longino A Martin K Leyba K. Prospective evaluation of venous excess ultrasound for estimation of venous congestion. Chest 2024; 165:590–600. 37813180 10.1016/j.chest.2023.09.029 PMC11317813

[60] Trigkidis KK Siempos II Kotanidou A. Early trajectory of venous excess ultrasound score is associated with clinical outcomes of general ICU patients. Shock 2024; 61:400–405. 38517247 10.1097/SHK.0000000000002321

[61] Ruste M Sghaier R Chesnel D. Perfusion-based deresuscitation during continuous renal replacement therapy: a before-after pilot study (The early dry Cohort). J Crit Care 2022; 72:154169. 36201978 10.1016/j.jcrc.2022.154169

[62▪] Bagshaw SM Neyra JA Tolwani AJ. Debate: intermittent hemodialysis versus continuous kidney replacement therapy in the critically ill patient: the argument for CKRT. Clin J Am Soc Nephrol 2023; 18:647–660. 39074305 10.2215/CJN.0000000000000056 PMC10278790

[63▪] De Waele JJ Boelens J. Antimicrobial stewardship and molecular diagnostics: a symbiotic approach to combating resistance in the ED and ICU. Curr Opin Crit Care 2024; 30:231–238. 38525881 10.1097/MCC.0000000000001154

[64▪] Malbrain M Caironi P Hahn RG. Multidisciplinary expert panel report on fluid stewardship: perspectives and practice. Ann Intensive Care 2023; 13:89. 37747558 10.1186/s13613-023-01177-y PMC10519908

[65] Carr JR Hawkins WA Newsome AS. Fluid stewardship of maintenance intravenous fluids. J Pharm Pract 2022; 35:769–782. 33827313 10.1177/08971900211008261 PMC8497650

[66] Gamble KC Smith SE Bland CM. Hidden fluids in plain sight: identifying intravenous medication classes as contributors to intensive care unit fluid intake. Hosp Pharm 2022; 57:230–236. 35601708 10.1177/00185787211016339 PMC9117780

[67] Smith SE Smith LT Sikora A. Relationship between medication regimen complexity and pharmacist engagement in fluid stewardship. Am J Health Syst Pharm 2024. zxae369. 39657137 10.1093/ajhp/zxae369

[68▪] Schortgen F Tabra Osorio C Carpentier D. Fluid intake in critically ill patients: the ‘save useless fluids for intensive resuscitation’ multicenter prospective cohort study. Crit Care Med 2024; 52:258–267. 37909832 10.1097/CCM.0000000000006091

[69▪] Biesheuvel LA Dongelmans DA Elbers PWG. Artificial intelligence to advance acute and intensive care medicine. Curr Opin Crit Care 2024; 30:246–250. 38525882 10.1097/MCC.0000000000001150 PMC11064910

[70] Vega SJ Berard D Avital G. Adaptive closed-loop resuscitation controllers for hemorrhagic shock resuscitation. Transfusion 2023; 63: (Suppl 3): S230–S240. 37071780 10.1111/trf.17377

